# Rescue Needle Knife Papillotomy Endoscopic Retrograde Cholangiopancreatography (ERCP) Cannulation: Success and Complications in a Retrospective Comparative Study With Procedure Time-Matched Controls

**DOI:** 10.7759/cureus.90141

**Published:** 2025-08-15

**Authors:** Byung Hyo CHA, Myoung-jin Jang, Osama M Idris, Said Hassan

**Affiliations:** 1 Gastroenterology, Sheikh Khalifa Specialty Hospital, Ras Al Khaimah, ARE; 2 Gastroenterology, Seoul National University Hospital, Seoul, KOR; 3 Medical Research Collaborating Center, Seoul National University Hospital, Seoul, KOR; 4 Surgery, Ras Al Khaimah Medical and Health Science University, Ras Al Khaimah, ARE

**Keywords:** difficult biliary cannulation, endoscopic retrograde cholangio-pancreatography, endoscopic sphincterotomy, needle knife, post-ercp complications, success rates

## Abstract

Background: Endoscopic retrograde cholangiopancreatography (ERCP) is a crucial endoscopic procedure for pancreato-biliary diseases for diagnostic and therapeutic purposes. Although commonly performed, difficult biliary cannulation (DBC) cases remain challenging when using conventional technology alone.

Objective: This study aimed to assess the efficacy and safety of rescue needle knife papillotomy (RNKP) compared to sphincterotomy only (SPTO) in DBC.

Methods: A retrospective observational study with sequential intervention analysis to evaluate outcomes between the SPTO and RNKP groups among endoscopic sphincterotomy (EST)-naïve cases that underwent ERCP in Sheikh Khalifa Specialty Hospital in the UAE. RNKP was performed after an unsuccessful conventional cannulation attempt with SPTO, utilizing the delicate technique of a needle knife catheter. Comparative analysis of procedural outcomes and adverse events was conducted using the procedure time-matched case pairs of both groups.

Results: Among 333 EST-naïve cases, SPTO was attempted initially in all patients, achieving successful cannulation in 277 (83.2%). In 56 cases where SPTO failed, the RNKP technique was applied, resulting in 55 successful cannulations with only one overall failure, leading to a total cannulation success rate of 332 (99.1%). After 1:1 time-matched case-control selection, 43 case-control pairs of SPTO vs. RNKP groups were analyzed. There were no statistically significant differences in post-ERCP complications between the two groups: cholangitis occurred in 2 cases (5.6%) in the SPTO group vs. 3 (7.3%) in the RNKP group (p=0.855); pancreatitis occurred in 1 (2.3%) vs. 3 (7.0%) (p=0.609); bleeding occurred in 0 (0%) vs. 1 (2.3%); and perforation did not occur in either group (0%).

Conclusion: RNKP is a highly effective rescue technique for difficult biliary cannulation, significantly increasing success rates following failed conventional SPTO cannulation, without elevating complication risks. This approach offers valuable clinical utility, especially for early-career endoscopists managing complex ERCP cases.

## Introduction

Endoscopic retrograde cholangiopancreatography (ERCP) is a widely used, minimally invasive procedure that plays a critical role in diagnosing and treating a variety of pancreato-biliary diseases. One of the key steps in this complex procedure is selective cannulation, which is essential for initiating any further diagnostic or therapeutic interventions. Even among experienced endoscopists, up to 15-30% of initial ERCP attempts may fail when using standard cannulation methods alone [1].

To address these challenges, several alternative techniques have been developed over the past few decades. These include needle knife precut, fistulotomy, and other advanced methods aimed at improving cannulation success in cases where conventional techniques fail. However, reported success rates for these advanced techniques vary widely across studies, reflecting differences in operator experience, patient characteristics, and procedural approaches [2,3].

Among these methods, needle knife papillotomy (NKP) has emerged as a modified technique of needle knife fistulotomy (NKF). NKP involves a needle knife incision on the surface of the papilla from the ampulla of Vater (AOV) orifice [4]. The aim of this study is to compare the outcomes and complication rates of standard sphincterotomy with the rescue needle knife papillotomy (NKP) technique in cases of difficult cannulation.

## Materials and methods

Study design and participants

We conducted a retrospective comparative study with sequential intervention analysis to evaluate the success rate and adverse events of rescue needle knife papillotomy (RNKP) versus sphincterotomy only (SPTO) during endoscopic retrograde cholangiopancreatography (ERCP) procedures. This study included patients who underwent ERCP at a tertiary referral center, Sheikh Khalifa Specialty Hospital in the UAE. The data were collected retrospectively from the medical records between Jan 2016 and Dec 2021. The study was approved by the Research Ethics Committee of the Ministry of Health and Prevention, UAE, with reference No: MOHAP/DXB-REC/JJJ/No.68/2022. Informed consent was waived due to the retrospective nature of the study, with all patient data anonymized and de-identified. Patients who underwent ERCP procedures during the specified period were considered eligible. Excluded from the study were patients undergoing ERCP with stent revision or removal, cases with gastric outlet obstruction, anatomical variations, endoscopic ultrasound (EUS) combined procedures without papillary cannulation, those with severe benign biliary strictures, and those requiring endoscopic papillectomy. Additionally, patients with immature termination due to anesthesia complications were also excluded.

Procedure

All the procedures were performed under fluoroscopic guidance by an experienced endoscopist with expertise in therapeutic ERCP, having performed more than 2,000 cases. The patients were positioned prone, switching to the left lateral decubitus position when necessary, under general anesthesia. Continuous monitoring of vital signs, oxygen saturation, ECG, and capnography was conducted throughout the procedure, ensuring a high level of proficiency and minimizing procedural risks.

ERCP was performed using the Olympus TJF-260V duodenoscope (Olympus, Tokyo, Japan), with the scope inserted through the oral cavity and gently advanced to the second portion of the duodenum, reaching the ampulla of Vater orifice precisely and safely. Cannulation was initiated with a conventional sphincterotomy using the D.A.S.H™ Sphincterotome (Cook Medical Europe Ltd.) and the Visiglide II (Olympus, Tokyo, Japan) guidewire.

**Figure 1:**
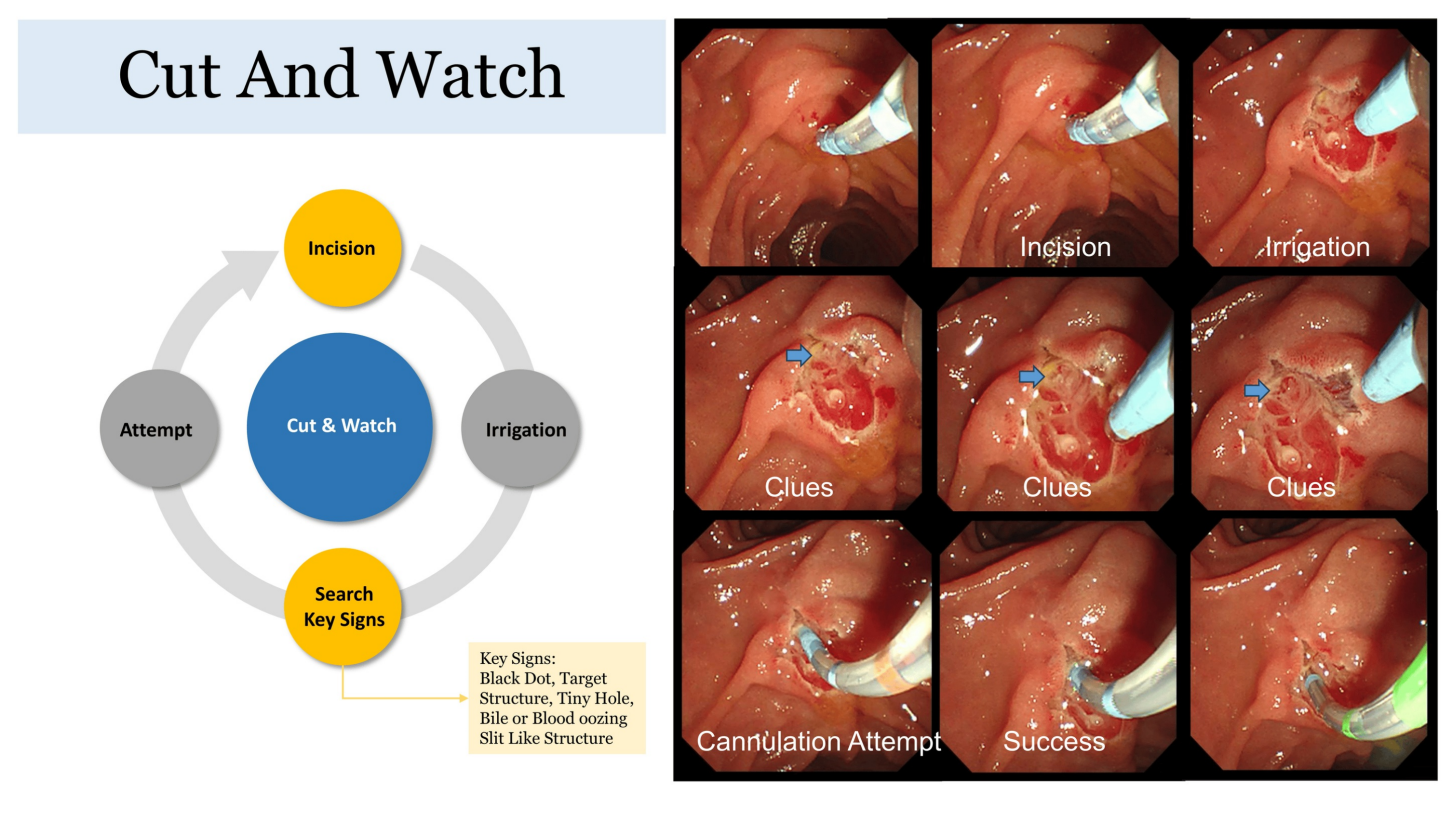
Key steps in the needle knife papillotomy (NKP) "cut and watch" technique. This figure, derived from a representative case included in the current study, illustrates the iterative "Cut and Watch" approach—a methodical technique for achieving biliary duct cannulation. The process begins with an initial incision, followed by irrigation to improve visualization. The endoscopist then searches for key visual clues, such as a black dot, target structure, slit-like opening, or bile fluid oozing, indicating bile duct exposure (blue arrows). Once such features are identified, a cannulation attempt is made. If unsuccessful, additional incremental incisions are performed, repeating the cycle to ensure safe and effective cannulation.

If conventional sphincterotomy was unsuccessful, the procedure shifted to needle knife papillotomy based on the definition of difficult biliary cannulation (DBC), which is defined as requiring more than 5-20 minutes for cannulation, more than five attempts at cannulation, or more than three inadvertent pancreatic duct cannulations [5]. In such cases, the Microknife™ XL Triple-Lumen Needle Knife (Boston Scientific, USA) was used to perform an electrical incision from the ampulla of Vater’s orifice to the 11 o'clock direction up to the major folds of the major papilla (Figure 1).

**Figure 2:**
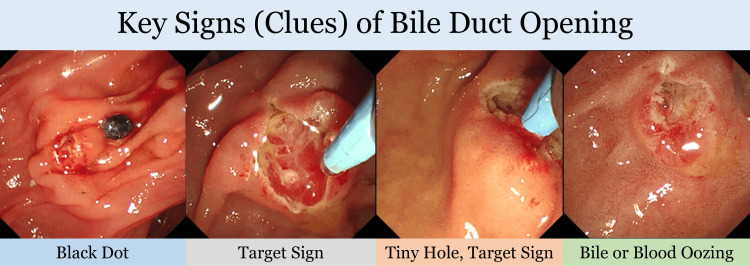
Suggested visual clues for identifying bile duct opening during the "cut and watch" technique. This figure is taken from a representative case included in this study and highlights potential visual clues that may assist in identifying the bile duct opening during the "Cut and Watch" approach. Features observed include a possible black dot (panel 1), a target-like appearance (panel 2), a tiny hole resembling a target sign (panel 3), and fluid suggestive of bile or blood oozing (panel 4). These clues, identified through video review of the procedure, are intended to guide clinicians during the iterative incision–irrigation–observation process.

The "cut and watch" technique was employed as needed, involving incremental cuts followed by observation to assess bile duct exposure and facilitate safe cannulation. The process included irrigation to wash the area and searching for signs of bile duct opening, such as black dots, tiny holes, target signs, slit-like appearances, or bile fluid oozing for a minimum of 1-2 minutes. Once the bile duct opening was identified, an attempt was made to cannulate it. Additional cuts were made as necessary, following the same meticulous approach each time (Figure 2).

Primary and secondary outcomes

The primary outcome was the overall biliary cannulation success rate, defined as successful deep biliary cannulation using initial SPTO or subsequent RNKP techniques during ERCP. The secondary outcomes were defined as the incidence of procedure-related adverse events, specifically post-ERCP hyperamylasemia, cholangitis, pancreatitis, bleeding, and perforations. Post-ERCP hyperamylasemia was defined as a serum amylase level exceeding three times the upper normal limit (UNL) within 24 hours of the procedure, without accompanying abdominal pain or clinical features of pancreatitis. Post-ERCP pancreatitis (PEP) was defined according to the Cotton criteria as new or worsened abdominal pain consistent with pancreatitis, combined with serum amylase levels elevated to more than three times the UNL at 24 hours post-procedure, and requiring unplanned hospital admission or a prolonged hospitalization of more than two days [6]. Post-ERCP cholangitis (PEC) was diagnosed based on the Tokyo Guidelines 2018, requiring clinical signs of infection such as fever and leukocytosis, along with evidence of cholestasis, including jaundice or elevated bilirubin or alkaline phosphatase, and imaging findings consistent with biliary obstruction or dilation occurring after the procedure [7]. Post-ERCP bleeding (PEB) was considered clinically significant if it necessitated endoscopic hemostasis or blood transfusion or resulted in a hemoglobin drop of more than 2 g/dL. Post-ERCP perforation was defined as radiologic or endoscopic evidence of gastrointestinal wall disruption requiring medical or surgical intervention, such as retroperitoneal air with clinical symptoms, free intraperitoneal air, or contrast extravasation.

Statistical analysis

To minimize the impact of temporal variability in operator experience and procedural practices over the study period, cases in the RNKP group were matched 1:1 with controls from the SPTO group based on procedure time. Variables collected included demographic data, body mass index (BMI), hospital stay, cumulative radiation exposure, ERCP time, cannulation time, needle knife time, pain scores, American Society of Anesthesiology (ASA) classification, diabetes, hypertension, endoscopic retrograde biliary drainage (ERBD) insertion, PD-related interventions, and post-procedure complications. Data were analyzed using R statistics software version 4.3.2 and Microsoft Excel 365 (Redmond, USA). For the unmatched groups, continuous variables were expressed as mean ± standard deviation (SD) and compared using the student’s t-test, while categorical variables were analyzed using the chi-square test or Fisher’s exact test where appropriate. Statistical significance was set at p < 0.005.

To ensure comparability between the RNKP group and the SPTO group, cases were matched in a 1:1 nearest matching fashion based on procedure time, using the matchit() function in R statistics software. Following the matching process, we used paired t-tests for continuous variables and McNemar’s test for categorical variables to compare the matched groups.

## Results

**Figure 3:**
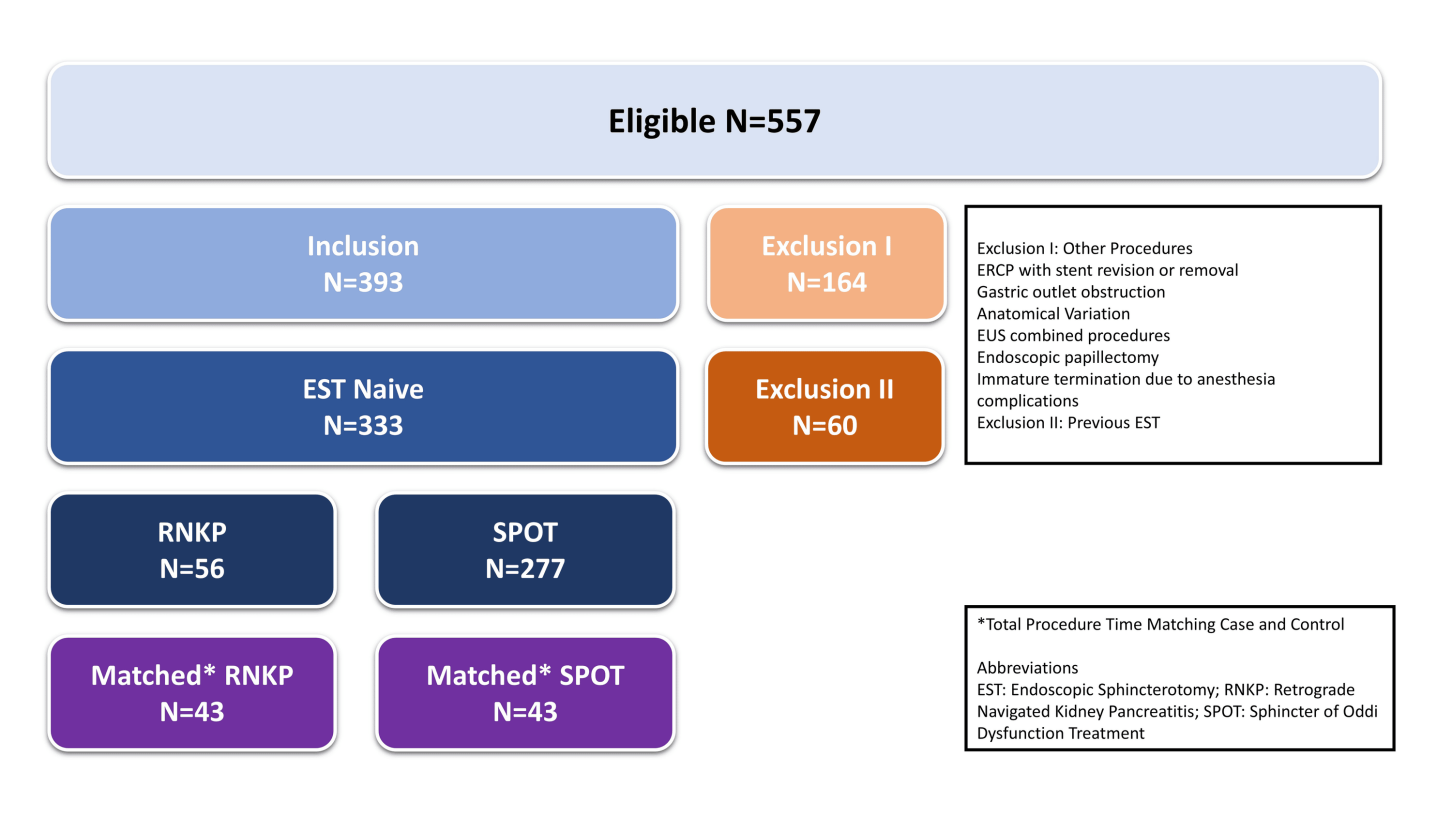
Enrollment process of study participants. EST: Endoscopic Sphincterotomy; RNKP: Retrograde Navigated Kidney Pancreatitis; SPTO: Sphincter of Oddi Dysfunction Treatment. The image is created by the author.

Of the 557 participants eligible for the study, 333 EST-naïve cases were included in the final analysis. A total of 224 cases were excluded due to various criteria, including previous EST, gastric outlet obstruction, anatomical variations, and other miscellaneous irrelevant procedural challenges. The entire enrollment process is depicted in Figure 3.

Baseline characteristics

As shown in Table 1, there were no statistically significant differences in demographic characteristics between the two groups, including age, sex, body mass index (BMI), and American Society of Anesthesiology (ASA) physical status classification scores.

**Table 1:** Comparison of demographic characteristics, procedure-related variables, and complication rates between the two groups before the procedure time matching. Data are presented as number (percentage) or mean ± standard deviation for continuous variables and number (percentage), n (%), for categorical variables. P-value¹ represents statistical comparisons between the SPTO and RNKP groups before matching, while P-value² refers to comparisons between 1:1 time-matched groups. P-value¹ and Test Stat^1^ refer to comparisons between the SPTO and RNKP groups before matching, calculated using independent t-tests for continuous variables and chi-square or Fisher’s exact tests for categorical variables. BMI: Body mass index, ERBD: Endoscopic retrograde biliary drainage, ERCP: Endoscopic retrograde cholangiopancreatography, PD: Peritoneal dialysis

Contents	Scale	SPTO N=277	RNKP N=56	Total N=333	p-value¹	Test Stat^1^
Age (Years)	Mean ± SD	50.2 ± 19.3	52.8 ± 20.5	50.2 ± 19.3	0.146	t = -0.87
Gender	Female, N (%)	148 (70.5%)	29 (51.8%)	177 (66.5%)	0.985	χ² = 0.01
	Male, N (%)	62 (29.5%)	27 (48.2%)	89 (33.5%)		
BMI (kg/m^2^)	Mean ± SD	28.9 ± 8.1	28.3 ± 6.6	28.9 ± 8.1	0.204	t = 0.60
Comorbidity	ASA score=1, N (%)	108 (34.1%)	16 (29.6%)	124 (37.2%)	NA	NA
	ASA score=2, N (%)	125 (39.4%)	25 (46.3%)	150 (45.4%)		
	ASA score=3, N (%)	73 (23.0%)	11 (20.4%)	94 (25.2%)		
	ASA score=4, N (%)	11 (3.5%)	2 (3.7%)	13 (3.9%)		
ERBD Stent Insertion	Yes, N (%)	200 (72.2%)	45 (80.3%)	245 (73.6%)	0.350	χ² = 1.20
Total Radiation Exposer Time (Min)	Mean ± SD	8.94 ± 7.4	9.9 ± 6.4	8.9 ± 7.4	0.134	t = -1.08
Total Procedure Time (ERCP, Min)	Mean ± SD	36.6 ± 22.7	49.5 ± 24.6	36.6 ± 22.7	0.003	t = -3.63
Total Cannulation Time (Min)	Mean ± SD	5.9 ± 11.2	22.0 ± 15.3	5.9 ± 11.2	< 0.01	t = -7.45
Total Needle Knife Time (Min)	Mean ± SD	12.3 ± 11.1	12.4 ± 11.3	12.3 ± 11.2	0.778	t = -0.08
PD Cannulation by Catheter	Yes, N (%)	81 (29.2%)	30 (50.8%)	111 (32.4%)	0.001	χ² = 6.81
PD Cannulation by Guidewire	Yes, N (%)	76 (27.4%)	26 (46.4%)	102 (30.6%)	0.023	χ² = 7.04
PD Contrast Injection	Yes, N (%)	42 (15.2%)	26 (46.4%)	68 (15.2%)	0.008	χ² = 26.13
PD Stent Insertion	Yes, N (%)	4 (1.4%)	4 (7.1%)	8 (2.4%)	0.044	χ² = 4.25
Post-ERCP Hyperamylasaemia	Yes, N (%)	50 (19.8%)	15 (30.6%)	65 (30.6%)	0.140	χ² = 2.80
Post-ERCP Cholangitis	Yes, N (%)	14 (6.5%)	3 (6.5%)	17 (6.5%)	0.989	χ² = 0.24
Post ERCP Pancreatitis	Yes, N (%)	15 (5.9%)	4 (7.8%)	19 (5.9%)	0.938	χ² = 0.01
Post-ERCP Bleeding	Yes, N (%)	6 (2.4%)	2 (3.9%)	8 (2.4%)	0.941	χ² = 0.00
Post-ERCP Perforation	Yes, N (%)	1 (0.4%)	0 (0.0%)	1 (0.4%)	0.816	χ² = 0.00

Cannulation success rates

Standard cannulation using the SPTO technique was attempted in all 333 EST naïve patients, with successful biliary access achieved in 277 patients (83.2%). In 56 cases where SPTO failed, the RNKP technique was applied, resulting in 55 additional successful cannulations. Two cases were not tried after SPTO cannulation because of severe strictures, and one RNKP case failed due to incidental failure of electrosurgical unit. This yielded an overall cannulation success rate of 330 among 333 (99.1%). Notably, the application of RNKP after SPTO attempts increased the cannulation success rate by 15.9%.

Procedure metrics and complications in unmatched analysis

In the unmatched analysis, the RNKP group had significantly longer procedure times and cannulation durations compared to the SPTO group (p = 0.003 and p < 0.001, respectively), consistent with the complexity of difficult cannulation cases. However, there were no statistically significant differences between the groups in terms of complication rates, including radiation doses, total radiation times, hyper-amylasemia/lipasemia, post-ERCP pancreatitis (PEP), bleeding, and perforation (Table 1).

Adverse outcome comparison in matched groups

After observing a significant difference in procedure time between the two groups prior to matching, we performed 1:1 matching based on procedure time to reduce its potential confounding effect on outcome comparisons. Following matching, no significant differences were observed between the RNKP and SPTO groups in terms of key procedure-related parameters including radiation exposure, biliary stent use, unintended pancreatic duct cannulation attempts. Importantly, complication profiles remained comparable, with similar rates of hyperamylasemia/hyperlipasemia, PEP, bleeding and perforation observed in both groups (Table 2).

**Table 2:** Comparison of demographic characteristics, procedure-related variables, and complication rates between the two groups after the procedure time matching. Data are presented as number (percentage) or mean ± standard deviation for continuous variables and number (percentage), n (%), for categorical variables. P-value¹ represents statistical comparisons between the SPTO and RNKP groups before matching, while P-value² refers to comparisons between 1:1 time-matched groups. P-value² and Test Stat^2^ refer to comparisons after 1:1 matching based on procedure time, using paired t-tests for continuous variables and McNemar’s test for categorical variables. BMI: Body mass index, ERBD: Endoscopic retrograde biliary drainage, ERCP: Endoscopic retrograde cholangiopancreatography, PD: Peritoneal dialysis

Contents	Scale	Matched SPTO N=43	Matched RNKP N=43	Matched Total N=86	p-value²	Test Stat^2^
Age (Years)	Mean ± SD	52.0 ± 19.1	51.9 ± 20.0	52.0 ± 19.5	0.98	t = 0.02
Gender	Female, N (%)	21 (48.8%)	24 (55.8%)	45 (52.3%)	0.67	χ² = 0.19
	Male, N (%)	22 (51.2%)	19 (44.2%)	41 (47.7%)		
BMI (kg/m^2^)	Mean ± SD	30.5 ± 9.9	29.6 ± 6.7	30.1 ± 8.4	0.61	t = 0.49
Comorbidity	ASA score=1, N (%)	10 (23.3%)	15 (34.9%)	25 (29.1%)		
	ASA score=2, N (%)	19 (44.2%)	19 (44.2%)	38 (44.2%)		
	ASA score=3, N (%)	12 (27.9%)	8 (18.6%)	20 (23.3%)		
	ASA score=4, N (%)	2 (4.7%)	1 (2.3%)	3 (3.5%)		
ERBD Stent Insertion	Yes, N (%)	33 (76.7%)	35 (81.4%)	68 (79.1%)	0.79	χ² = 0.07
Total Radiation Exposer Time (Min)	Mean ± SD	8.9 ± 7.4	9.7 ± 8.3	9.3 ± 5.8	0.78	t = 0.30
Total Procedure Time (ERCP, Min)	Mean ± SD	36.6 ± 22.7	37.9 ± 22.4	43.6 ± 22.3	0.47	t = -1.19
Total Cannulation Time (Min)	Mean ± SD	5.9 ± 11.2	14.5 ± 16.0	19.5 ± 16.3	0.15	t = -1.44
Total Needle Knife Time (Min)	Mean ± SD	12.3 ± 11.1	11.1 ± 9.6	10.6 ± 12.1	0.94	t = 0.18
PD Cannulation by Catheter	Yes, N (%)	23 (53.5%)	20 (46.5%)	43 (50.0%)	0.67	χ² = 0.19
PD Cannulation by Guidewire	Yes, N (%)	21 (48.8%)	17 (39.5%)	38 (44.2%)	0.52	χ² = 0.42
PD Contrast Injection	Yes, N (%)	15 (34.9%)	16 (37.2%)	31 (36.0%)	1.00	χ² = 0.00
PD Stent Insertion	Yes, N (%)	0 (0.0%)	1 (2.3%)	1 (1.2%)	1.00	χ² = 0.00
Post-ERCP Hyperamylasaemia	Yes, N (%)	9 (21.4%)	11 (27.5%)	20 (24.4%)	0.70	χ² = 0.52
Post-ERCP Cholangitis	Yes, N (%)	2 (5.6%)	3 (7.3%)	5 (6.5%)	0.99	χ² = 0.77
Post-ERCP Pancreatitis	Yes, N (%)	1 (2.3%)	3 (7.0%)	4 (4.7%)	0.61	χ² = 0.26
Post-ERCP Bleeding	Yes, N (%)	0 (0.0%)	1 (2.3%)	1 (1.2%)	NA	NA
Post-ERCP Perforation	Yes, N (%)	0 (0.0%)	0 (0.0%)	0 (0.0%)	NA	NA

Visual indicators for bile duct orifice identification

Through retrospective review of all available procedure video recordings, we observed repeated patterns of visual features that can assist in localizing the bile duct opening during RNKP. These included a small black dot, a target sign, narrow slit-like structures, and intermittent bile or blood-stained fluid oozing. These signs were frequently noted in successful cannulation and are summarized in Figure 2.

## Discussion

The ERCP procedure is indispensable yet inherently risky, with biliary cannulation being a key determinant of success and complications. A multinational, prospective study using data from a web-based ERCP Quality Network registry, encompassing over 13,000 procedures, reported an 89.8% biliary cannulation success rate with conventional techniques, emphasizing the importance of optimizing strategies to improve outcomes [8]. Many society guidelines, including ESGE, ASGE, and JGES, recommend changing to alternative techniques such as guidewire-assisted cannulation, double-guidewire technique, or precut sphincterotomy within 5-10 minutes or after multiple inadvertent pancreatic duct cannulations to avoid complications like post-ERCP pancreatitis [2,9,10].

Alternative methods for difficult biliary cannulation have demonstrated varying levels of success and complication rates. In the systematic review and network meta-analysis by Facciorusso et al., trans-pancreatic sphincterotomy increased the biliary cannulation success rate by 29% compared to standard techniques (risk ratio [RR], 1.29; 95% confidence interval [CI], 1.05-1.59) and had a lower post-ERCP pancreatitis (PEP) rate than the pancreatic guidewire-assisted technique (RR, 0.53; 95% CI, 0.30-0.92) [11]. Similarly, early needle-knife techniques improved the success rate by 47% compared to pancreatic stent-assisted techniques (RR, 1.47; 95% CI, 1.03-2.10) and reduced PEP rates by 39% compared to persistence with standard techniques (RR, 0.61; 95% CI, 0.37-1.00).

This study evaluated rescue needle-knife papillotomy performed immediately after failed conventional cannulation, a modified needle-knife precut technique, and found that it significantly increased the biliary cannulation success rate without increasing the risk of complications. This success is attributed to the "cut-and-watch" approach, which avoids premature cannulation attempts and emphasizes waiting until the bile duct stigmata are clearly visualized, allowing for precise and controlled cannulation with minimal complications.

Despite the absence of prophylactic measures against PEP in our study, we observed a low incidence of PEP, likely attributable to directing the needle-knife incision away from the pancreatic duct orifice and thus minimizing duct trauma, in contrast to techniques such as transpancreatic sphincterotomy or pancreatic guidewire-assisted methods, which have been considered more likely to increase the risk of pancreatic injury [12]. But Facciorusso et al. reported no significant difference in PEP rates between transpancreatic sphincterotomy and precut needle-knife techniques [11]. Therefore, other factors emphasized by their analysis-precise cannulation, minimizing mechanical injury, and shortening manipulation times-could collectively contribute to reducing PEP rates in our study [11,13].

Several studies, including those by Maharshi et al. [14] and Bapaye et al. [15], advocate for primary precut techniques, such as needle-knife fistulotomy (NKF) or needle-knife papillotomy (NKP), as an initial approach for difficult bile duct cannulation in ERCP, demonstrating significantly lower PEP rates (0.67% vs. 5.2%) and comparable cannulation success rates while minimizing repeated attempts at conventional cannulation. However, many studies suggest early rescue precut techniques reduce complications, including lower PEP rates and higher safety when performed by skilled endoscopists [16,17]. Furthermore, Udd et al. reported that sphincterotomy with guidewire achieves a success rate of over 97% without precut, underscoring the efficacy of conventional cannulation techniques [5]. Thus, the recommendation is to initiate biliary cannulation using primary sphincterotomy with a guidewire approach; an early precut technique, particularly NKP, should be employed to minimize procedural complications and prevent post-ERCP pancreatitis, keeping the 5-5-3 rules in mind, which means five minutes of cannulation time, five cannulation attempts, and three inadvertent pancreatic duct cannulations [2]. This strategy balances safety, efficiency, and success rates across diverse clinical scenarios.

The limitations of our study include the lack of a direct head-to-head comparison between conventional cannulation and needle-knife papillotomy (NKP), as well as a small sample size due to the limited number of NKP cases. Although the “5-5-1 rule” is often used to define difficult biliary cannulation, it is based on expert opinion and not supported by strong evidence or uniform guideline consensus. As a result, we could not apply this rule strictly in our retrospective study, which may have influenced the observed similarities in radiation exposure and cannulation time between groups. Additionally, the comparison between the RNKP and SPTO groups was limited by the lack of detailed stratification by factors such as age, gender, comorbidities, and cholangiographic findings, which may have provided deeper insights into patient-specific outcomes and enhanced the generalizability of our findings. Given that procedure time is closely related to difficult cannulation and is a straightforward metric to measure, we conducted a procedure time-matched case-control study to minimize its confounding effects, as highlighted by the ESGE Clinical Guideline [2]. However, due to the small size of the NKP case group, we were unable to account for many other confounding factors that might influence success rates and complication incidences, which limits the robustness of our findings.

## Conclusions

This study demonstrates that rescue needle knife papillotomy (RNKP), when performed with an effort to find the signs of proper bile duct orifice, such as “cut-and-watch” techniques, is an effective and safe method for achieving biliary access in case of difficult cannulation. By systematically observing subtle visual cues, such as black dots, target signs, tiny holes, or bile-stained fluid, endoscopists can more precisely identify the bile duct orifice and apply targeted, controlled incisions. Importantly, this technique did not increase the risk of post-procedural complications, even in technically complex cases. Beyond its clinical value, the structured and observational nature of the "cut-and-watch" approach offers a practical learning framework that can help ERCP beginners develop their skills more rapidly and confidently in managing challenging cannulation scenarios.
